# Calcineurin Subunits A and B Interact to Regulate Growth and Asexual and Sexual Development in *Neurospora crassa*

**DOI:** 10.1371/journal.pone.0151867

**Published:** 2016-03-28

**Authors:** Ranjan Tamuli, Rekha Deka, Katherine A. Borkovich

**Affiliations:** 1 Department of Plant Pathology and Microbiology, Institute for Integrative Genome Biology, University of California Riverside, Riverside, California, United States of America; 2 Department of Biosciences and Bioengineering, Indian Institute of Technology Guwahati, Guwahati, India; Georg-August-University of Göttingen Institute of Microbiology & Genetics, GERMANY

## Abstract

Calcineurin is a calcium/calmodulin dependent protein phosphatase in eukaryotes that consists of a catalytic subunit A and a regulatory subunit B. Previous studies in the filamentous fungus *Neurospora crassa* had suggested that the catalytic subunit of calcineurin might be an essential protein. We generated *N*. *crassa* strains expressing the A (*cna-1*) and B (*cnb-1*) subunit genes under the regulation of *P*_*tcu-1*_, a copper-responsive promoter. In these strains, addition of bathocuproinedisulfonic acid (BCS), a copper chelator, results in induction of *cna-1* and *cnb-1*, while excess Cu^2+^ represses gene expression. Through analysis of these strains under repressing and inducing conditions, we found that the calcineurin is required for normal growth, asexual development and female fertility in *N*. *crassa*. Moreover, we isolated and analyzed *cnb-1* mutant alleles generated by repeat-induced point mutation (RIP), with the results further supporting roles for calcineurin in growth and fertility in *N*. *crassa*. We demonstrated a direct interaction between the CNA-1 and CNB-1 proteins using an assay system developed to study protein-protein interactions in *N*. *crassa*.

## Introduction

Calcineurin is the only serine/threonine protein phosphatase that depends on calcium/calmodulin (Ca^2+^/CaM) [1, 2]. The heterodimeric calcineurin protein consists of a catalytic subunit (A) that binds to calcium sensor CaM, and a regulatory subunit (B) that contains four Ca^2+^-binding domains [1–4]. CaM binds to the regulatory domain of the catalytic subunit in response to the cytosolic Ca^2+^concentration, and stimulates phosphatase activity that converts signals to various outputs by dephosphorylating target proteins [5, 6]. The functions of calcineurin have been studied in various organisms, including mammals, invertebrates, plants, parasites, and fungi [4]. The well-studied targets of calcineurin are the transcription factor Nuclear Factor of Activated T cells (NFAT) in mammals and the calcineurin responsive zinc-finger 1 (Crz1) in fungi, which upon dephosphorylation by activated calcineurin, are localized to the nucleus to regulate expression of target genes [7].

In mammals, the NFAT transcription factors are essential for several processes during organogenesis, including endocrine, immune, nervous, respiratory, skeletal and vascular systems [8, 9]. Defective calcineurin pathways have been linked to diseases such as cancer, cardiac hypertrophy, diabetes, Down’s syndrome and other degenerative brain diseases in humans [10]. Calcineurin is essential for the development of *Drosophila melanogaster*, in that flies with a mutation in the calcineurin B2 gene die at the late larval or early pupal stage [11]. In the worm *Caenorhabidits elegans*, calcineurin controls sensitivity to odorants, and may play a role in either motor neuron or muscle development [12]. In plants, the calcineurin B-like proteins (CBLs) are calcium sensors that regulate the activity of the CBL-interacting protein kinase (CIPK) and CBL-CIPK pathways function in signaling processes including environmental stress responses, nutrient sensing and adaptation [13]. In the malaria parasite *Plasmodium falciparum*, the chaperone heat shock protein 90 (Hsp90) associates with calcineurin, in a process that is necessary for erythrocytic replication of the parasite in the host [14].

In fungi, calcineurin has been mainly studied in the budding yeast *Saccharomyces cerevisiae* and certain fungal pathogens. In *S*. *cerevisiae*, the catalytic subunit is encoded by the *CNA1/CMP1* and *CNA2/CMP2* genes, while the regulatory subunit is encoded by the *CNB1* gene; all three calcineurin subunits are individually or collectively non-essential for growth [15–17]. Calcineurin in *S*. *cerevisiae* is involved in the pheromone response and tolerance to high ion stress [15–18]. In *Candida albicans*, calcineurin subunits *CNA* and *CNB* are not essential, but play roles in cation homeostasis, and are essential for survival during membrane stress, in serum-containing media, and for virulence in a mouse model of systemic infection [19, 20]. In the opportunistic human fungal pathogen *Cryptococcus neoformans*, *CNA1* and *CNB1* are essential for fungal virulence and for growth at 37°C [21, 22]. In addition, calcineurin also regulates hyphal elongation and mating in *C*. *neoformans* [23]. In the rice blast fungus, *Magnaporthe oryzae*, the calcineurin catalytic subunit *MCNA* and the *CNB*-like genes play a role in infection-related differentiation and pathogenicity [24, 25]. In *Mucor circinelloides*, the causative zygomycete of human mucormycosis, there are three catalytic A subunit genes, *cnaA*, *cnaB*, and *cnaC*, and one regulatory gene, *cnbR* [26]. The calcineurin pathway plays a key role in dimorphic transitions and virulence in *M*. *circinelloides* [26]. In *Ustilago maydis*, a dimorphic basidiomycete that causes corn smut disease, the *ucn1* catalytic subunit regulates morphogenesis and pathogenesis [27].

In the filamentous fungus *Neurospora crassa*, the calcineurin catalytic subunit *cna-1* has been suggested to be an essential gene, and knock-down experiments using *cna-1* antisense RNA revealed its requirement for normal hyphal branching, growth, and maintenance of the apical tip-high Ca^2+^ gradient [28]. In addition, analysis of insertional and repeat-induced point mutation (RIP) [29] mutants of the calcineurin regulatory subunit B, *cnb-1*, suggested requirements for normal vegetative growth in *N*. *crassa* [30]. Moreover, it has been demonstrated that CNB-1 binds to the calcineurin-dependent response element (CDRE) in the copper-induced metallothionein (CuMT) gene, suggesting a putative role for calcineurin in the regulation of CuMT in *N*. *crassa* [31]. Since homokaryotic knockout mutants of the essential calcineurin catalytic subunit A are not available, and no experiments have investigated its function using temperature-sensitive alleles or regulatable promoters, information regarding detailed functions of calcineurin in *N*. *crassa* have remained elusive.

In *N*. *crassa*, the *tcu-1* gene encodes a high affinity copper transporter and its expression is precisely controlled by copper availability [32]. In addition, the kinetics of induction and repression of heterologous genes under the *tcu-1* promoter (P_*tcu-1*_) are rapid and stable [33]. We therefore used this system to investigate functions of *cna-1* and *cnb-1* by generating *N*. *crassa* strains with these genes under the control of *P*_*tcu-1*_. We found that calcineurin is required for normal growth, asexual development, and sexual fertility in *N*. *crassa*. These findings were supported by results from analysis of *cnb-1* mutant alleles generated by RIP. In addition, we demonstrated a direct interaction between the CNA-1 and CNB-1 proteins using an assay system developed to study protein-protein interactions in *N*. *crassa* [34].

## Materials and Methods

### Media, Growth Conditions, and Transformation Procedure

*Neurospora crassa* wild type strains 74-OR23-IVA and ORS-SL6a and all other strains were either obtained from the Fungal Genetics Stock Center (FGSC, Manhattan, KS) or generated in our laboratory (Table 1). Strains were cultured on Vogel’s minimal medium (VM) [35] to support vegetative growth, while sexual development was induced using synthetic crossing medium (SCM) [36]. Conidia used for inoculating cultures were propagated in 13x100 mm glass tubes containing VM with 1% agar, and grown for 3 days at 30°C in the dark and for 4 days at 25°C in the light [37, 38]. The growth of *N*. *crassa* strains was measured essentially as described previously [39–41]. Sorbose-containing medium (FGS) was used for ascospore germination and isolation of transformant colonies [42]. VM with proline as the nitrogen source was used for selection with Ignite [43]. When indicated, the growth medium was supplemented with pantothenate (Product number P2250; Sigma-Aldrich, St. Louis, MO), hygromycin (Calbiochem; San Diego, CA), Ignite (extracted from Finale; Farnam Companies INC.; Phoenix, AZ) [44, 45], or Nourseothricin (clonNAT; WERNER BioAgents; Germany) at a concentration of 10, 220, 400, and 50 μg/ml, respectively. Stocks of pantothenate and inositol were prepared and used as described previously [46; http://www.fgsc.net/methods/stanford.html]. The standard VM contains 50 mM CuSO_4_; where indicated, bathocuproinedisulfonic acid (BCS; Catalog number 164060010, Acros Organics, Geel, Belgium) and copper II sulfate (CuSO_4_; Catalog number 830521, Chempure, Texas, USA) were added to VM at the indicated concentrations. All sequencing was performed at the Genomics Core, Institute for Integrative Genome Biology, UC Riverside. *E*. *coli* strain DH5α [47] was the recipient for plasmid transformations. Transformation of *N*. *crassa* strains was performed using an Eppendorf Electroporator 2510 at a setting of 2000 volts as described previously [48].

**Table 1:** *Neurospora crassa* strains used in this study.

Strain	Genotype	Reference
74-OR23-IVA	Wild type; *mat A*	FGSC 2489
ORS-SL6 a	Wild type; *mat a*	FGSC 4200
2074	*Δcnb-1*::*hph; mat a* (heterokaryon)	FGSC 2074
2173	*Δcna-1*::*hph; mat a* (heterokaryon)	FGSC 2173
51-4-1	Δ*rid-1*::*nat;* Δ*mus-51*::*nat; mat a*	^a^Ouyang et al., submitted
T-51-4-1 (28)	Δ*rid-1*::*nat;* Δ*mus-51*::*nat; ∆pan-2*::P_*tcu-1*_::*cnb-1*::*v5*::*gfp; mat a* (heterokaryon)	This study
52-4-9	Δ*mus-52*::*nat*; *mat A*	FGSC 2479
T-52-4-9 (41)	Δ*mus-52*::*nat*; *∆cna-1*::*hph; ∆pan-2*::P_*tcu-1*_::*cna-1*::*V5*::*gfp; mat A* (heterokaryon)	This study
T-52-4-9 (632)	Δ*mus-52*::*nat*; *∆cna-1*::*hph; ∆inl*:: P_*ccg-1*_::*cna-1*::*S-tag*::*rfp; mat A* (heterokaryon)	This study
T-52-4-9 (679)	Δ*mus-52*::*nat*; *∆cnb-1*::*hph; ∆pan-2*::P_*ccg-1*_::*cnb-1*::*V5*::*gfp; mat A* (heterokaryon)	This study
2994	*∆pan-2*::P_*ccg-1*_::*V5*::*gfp; mat a*	^a^Ouyang et al., submitted
2995	*∆inl*:: P_*ccg-1*_::*S-tag*::*rfp; mat a*	^a^Ouyang et al., submitted
540	*∆cna-1*::*hph; ∆pan-2*::P_*tcu-1*_::*cna-1*::*V5*::*gfp; mat a*	This study
554	*∆pan-2*::P_*tcu-1*_::*cnb-1*::*V5*::*gfp; mat A*	This study
555	*∆pan-2*::P_*tcu-1*_::*cnb-1*::*V5*::*gfp; mat A*	This study
597	*∆cnb-1*::*hph; ∆pan-2*::P_*tcu-1*_::*cnb-1*::*V5*::*gfp; mat a*	This study
599	*∆cnb-1*::*hph; ∆pan-2*::P_*tcu-1*_::*cnb-1*^RIP^::*V5*::*gfp; mat A*	This study
600	*∆cnb-1*::*hph; ∆pan-2*:: P_*tcu-1*_::*cnb-1* ^RIP^::*V5*::*gfp; mat A*	This study
602	*∆cnb-1*::*hph; ∆pan-2*::P_*tcu-1*_::*cnb-1*^RIP^::*V5*::*gfp; mat A*	This study
727	*∆cnb-1*::*hph; ∆pan-2*::P_*ccg-1*_::*cnb-1*::*V5*::*gfp; mat a*	This study
767	*∆cna-1*::*hph; ∆inl*::P_*ccg-1*_::*cna-1*::*S-tag*::*rfp; mat a*	This study
CNB-1GFP+CNA-1RFP#5	Heterokaryon of strain 727 *+* strain 767	This study
GFP+RFP#7	Heterokaryon of strain 2994 *+* strain 2995	This study
CNB-1GFP+RFP#18	Heterokaryon of strain 727 *+* strain 2995	This study

^a^ Shouqiang Ouyang, Ilva E. Cabrera, Asharie J. Campbell and Katherine A. Borkovich, submitted.

### Construction of Strains Expressing Tagged Versions of *cna-1* or *cnb-1*

#### *∆cna-1*::*hph; ∆pan-2*::P_*tcu-1*_::*cna-1*::*V5*::*gfp; mat a* (strain 540)

The 2018 bp open reading frame (ORF) of calcineurin catalytic subunit A (NCU03804) was PCR-amplified using the primer pairs CNA-1-FOR and CNA-1-REV-GFP (Table 2), and gel purified. The *pan-2* (NCU10048.7) locus targeting vector pRS426PVGTCU1.5 (P_*tcu-1*_::*5xGly*::*V5*::*gfp*) [34] was linearized by restriction digestion with *Pac*I and *Sma*I (New England Biolabs, Ipswich, MA) and gel-purified. Approximately 100 ng each of the gel-purified linearized vector and the PCR product were joined in the yeast strain FY834 [49] using yeast recombinational cloning [50]. DNA from the transformed yeast was isolated using the yeast smash and grab method [51] and transformed into *Escherichia coli* DH5α ultra-competent cells [47]. One clone, pRTCNA-1(18), was confirmed by sequencing. The pRTCNA-1(18) vector was transformed into *N*. *crassa* strain 52-4-9 (Table 1). The initial transformants were selected on FGS plates supplemented with Ignite and pantothenate. A transformant strain, T-52-4-9 (41) (Table 1), was isolated and crossed with a heterokaryotic Δ*cna-1*::*hph; mat a* strain (2173; Table 1). Ascospores from this cross were germinated on FGS medium supplemented with pantothenate and BCS and then screened for resistance to hygromycin and Ignite, and pantothenate auxotrophy.

**Table 2:** Primers used in this study.

Primer	Sequence (5’→3’)
^a^CNA-1-FOR	CGCACACACATCCCCAACCAACCATGGAAAGCAACAATGGTACCGGCGC
^a^CNA-1-REV-GFP	GTTAGGGATAGGCTTTCCGCCGCCTCCGCCCGATCGCTTGCGGTCACTCAAC
^a^PAN2-PTCU1-0.5-FW	CCTTGCGTATATTCTGGACCGGTACACGGAACATCTCGTGAACAAGAAGG
CNA-1-5F	CTCTGAAAGAGGGCCTTGCC
^b^5HPHR	ATCCACTTAACGTTACTGAAATC
^a^CNB-1-FOR	CGCACACACATCCCCAACCAACCATGGGCAACACCACCAGCTCCGTCC
^a^CNB-1-REV-GFP	GTTAGGGATAGGCTTTCCGCCGCCTCCGCCGAATTGATCTGTTAAAGCGTCGACTGTC
CNB-1-5F	CACCACTTCCTCTCCATGTC
^a^CNA-1-pCCG-RFP-FW	CCACTTTCACAACCCCTCACATCAACCAAAATGGAAAGCAACAATGGTACCGGCGC
^a^CNA-1-pCCG-RFP-RV	AGCAGCGGTTTCTTTTCCGCCGCCTCCGCCCGATCGCTTGCGGTCACTCAAC
^a^CNB-1-pCCG-GFP-FW	CCACTTTCACAACCCCTCACATCAACCAAAATGGGCAACACCACCAGCTCCGTCC
PCCG-1-SEQ-FW	CCATCATCAGCCAACAAAGC
RFP-RV	TAGGGAGGTCGCAGTATCTG
GFP-RV	AACTCCAGCAGGACCATGTG

^a^ Sequences are from Ouyang, Cabrera, Campbell and Borkovich (submitted). See Materials and Methods for details.

^b^ Ref. [39]

We isolated a progeny with genotype *∆cna-1*::*hph; ∆pan-2*::P_*tcu-1*_::*cna-1*::*v5*::*gfp; mat a* (540; Table 1) that showed resistance to hygromycin, Ignite, and requirements for pantothenate and BCS. We verified the presence of the *cna-1* allele at the *pan-2* locus by PCR amplification with primers PAN2-PTCU1-0.5-FW and CNA-1-REV-GFP (Table 2), and further confirmed by sequencing. In addition, the *∆cna-1*::*hph* allele in strain 540 was verified by PCR using primers CNA-5F and 5HPHR (Table 2).

#### *∆cnb-1*::*hph; ∆pan-2*::P_*tcu-1*_::*cnb-1*::*V5*::*gfp; mat a* (strains 597, 599, 600, and 602)

The 1178 bp fragment of calcineurin regulatory subunit B (NCU03833) was PCR-amplified using the primer pairs CNB-1-FOR and CNB-1-REV-GFP (Table 2) and assembled with the *pan-2* locus targeting vector pRS426PVGTCU1.5 in yeast as described above. One clone, pRTCNB-1 (28), was confirmed by sequencing. The pRTCNB-1 (28) construct was transformed into the *N*. *crassa* strain 51-4-1 (Table 1). One transformant, T-51-4-1 (28), was first crossed with wild type *mat A* strain (FGSC 2489) and two homokaryotic progeny with the genotype *∆pan-2*::P_*tcu-1*_::*cnb-1*::*v5*::*gfp; mat A* (strains 554 and 555; Table 1) were isolated.

In order to isolate strains with the *cnb-1* gene under control of the *tcu-1* promoter in an otherwise *Δcnb-1*::*hph* background, the 554 and 555 strains were crossed with the *Δcnb-1*::*hph; mat a* strain (2074; Table 1). Ascospores from these crosses were germinated on medium supplemented with pantothenate and BCS and screened for resistance to hygromycin and Ignite, and pantothenate auxotrophy. We isolated three progeny (597, 599 and 600) from the cross of strain 554 to strain 2074 and one progeny (602) from the cross of strain 555 to strain 2074 of genotype *∆cnb-1*::*hph; ∆pan-2*::P_*tcu-1*_::*cnb-1*::*v5*::*gfp; mat a* (Table 1). The presence of the *cna-1* allele at the *pan-2* locus was verified by PCR amplification and further confirmed by sequencing (Genomics core, UCR). In addition, the *∆cnb-1*::*hph* allele in strain 597 was verified by PCR using primers CNB-1-5F and 5HPHR (Table 2).

#### *∆cna-1*::*hph; ∆inl*:: P_*ccg-1*_::*cna-1*::*S-tag*::*rfp; mat a* (767) and *∆cnb-1*::*hph;∆pan-2*::P_*ccg-1*_::*cnb-1*::*V5*::*gfp; mat a* (727) strains

The 2018 bp ORF of calcineurin subunit A (NCU03804) and the 1178 bp ORF of calcineurin subunit B (NCU03833) were PCR-amplified using the primer pairs CNA-1-pCCG-RFP-FW and CNA-1-pCCG-RFP-RV, and CNB-1-pCCG-GFP-FW and CNB-1-REV-GFP, respectively (Table 2). PCR products for the calcineurin subunits A and B were assembled, respectively, in the *inl* locus (NCU06666.7) targeting vector pRS426ISR (P_*ccg-1*_::*5xGly*::*S-tag*::*rfp*) and *pan-2* locus targeting vector pRS426PVG (P_*ccg-1*_::*5xGly*::*V5*::*gfp*) [34] that were linearized by restriction digestion with *Pac*I and *Sma*I (NEB). These fragments were assembled in yeast as described above, and pRTCNA-1 (6) and pRTCNB-1 (3) were isolated and confirmed by sequencing. The pRTCNA-1(6) and pRTCNB-1(3) constructs were transformed into *N*. *crassa* strain 52-4-9 (Table 1). The initial transformants were selected on VM supplemented with Ignite and pantothenate. Strains T-52-4-9 (632) and T-52-4-9 (679), transformed with the pRTCNA-1(6) and pRTCNB-1(3), respectively, were isolated and crossed with the heterokaryotic strains *∆cna-1*::*hph; mat a* (2173) and *∆cnb-1*::*hph; mat a* (2074), respectively. From these crosses, we isolated homokaryotic strains *∆cna-1*::*hph; ∆pan-2*::P_*ccg-1*_::*cna-1*::*S-tag*::*rfp; mat a* (767) and *∆cnb-1*::*hph; ∆pan-2*::P_*ccg-1*_::*cnb-1*::*V5*::*gfp; mat a* (727) (Table 1). The *cna-1* and *cnb-1* transgenes in the two strains were verified by PCR amplification with primer PCCG-1-SEQ-FW in combination with RFP-RV and GFP-RV, respectively (Table 2). In addition, the two strains (767 and 727) were resistant to hygromycin B and the presence of the *∆cna-1*::*hph* and the *∆cnb-1*::*hph* allele in the respective strains was confirmed, as described above for strains 540 and 597, respectively.

### Western Blot and Co-Immunoprecipitation Analysis

Strains were grown in liquid VM for 16 h as described in the figure legends and whole cell extracts were isolated essentially as described previously [52, 53]. Briefly, mycelia from these cultures were collected on a filter using vacuum, and then pulverized to a powder using a mortar and pestle in liquid nitrogen. Extraction buffer [50 mM HEPES (pH 7.7), 2 mM EGTA (pH 8.0), 2 mM EDTA (pH 8.0), 1% SDS, 10% glycerol, 100 mM NaCl, 1 mM Na_3_VO_4_ and 1 mM NaF] was added to the mycelial powder, and the samples were heated at 85°C for 5 min, after which 10 μl of 100 mM PMSF and 1 μl of fungal protease inhibitor cocktail (Product number P8215; Sigma-Aldrich, St. Louis, MO) were added. The crude protein extracts from these samples were isolated by centrifugation at 4000xg for 15 min at 4°C, and protein quantification was performed on the supernatants using the Bradford method (Protein Assay Dye Reagent Concentrate, Catalog number 500–0006; Bio-Rad, Hercules, CA). Samples containing 50 μg of protein were separated using a 10% SDS-PAGE gel and transferred onto a nitrocellulose membrane [54]. Membranes were probed using anti-GFP antibody at a dilution of (1:5000) (Catalog number A6455; Life Technologies; Carlsbad, CA) or anti S-tag antibody at a dilution of 1:3000 (Catalog number A190-135A; Bethyl Laboratories, Montgomery, TX) antibodies [54]. A horseradish peroxidase secondary antibody was used at a dilution of 1:2000 and bands were visualized by chemiluminescent detection as previously described [54]. In addition, after detecting via Western blotting, membranes were stained with an amido black solution (0.1% amido black from the Sigma-Aldrich Product number N3393, 10% acetic acid, 25% isopropanol) to show all proteins as an indication of protein transfer.

For co-immunoprecipitation analysis, forced heterokaryons (Table 1) were made by co-inoculating the individual strains in flasks containing VM agar and incubating at 30°C in the dark for 3 days and at room-temperature for four days under constant light. Conidia were isolated as described above and then inoculated in flasks containing 500 ml liquid VM at a concentration of 1x10^6^ conidia/ml. Flasks were incubated at 30°C for 16 h with shaking at 200 rpm. Cells were isolated by filtration through sterile shop towels and then ground using a mortar and pestle with liquid nitrogen. The powdered mycelia were transferred to a bead-beater (Biospec Products, Bartlesville, OK) containing glass beads and extraction buffer [50 mM Tris-Cl (pH 7.5), 1 mM EDTA, 6 mM MgCl_2_, 2.5 mM PMSF and 0.1% (v/v) of fungal protease inhibitor cocktail (Product number P8215; Sigma-Aldrich, St. Louis, MO)]. Cells were broken using three pulses of one minute each, with one minute intervals in between. The mixture was centrifuged at 16,000xg for 15 minutes at 4°C. The supernatant containing the whole cell extract was carefully transferred into a fresh tube and protein quantified using the Bradford assay (Bio-Rad, Hercules, CA). Then, an aliquot of 3 μl GFP-Trap agarose beads (Product numbergta-20; ChromoTek; GmbH, Germany) was taken in a microcentrifuge tube and beads were equilibrated by washing once with 1 ml of ice-cold solutions of 1X Tris-buffered saline with Tween-20 (TBST) [51] and twice with 1 ml of ice-cold extraction buffer by centrifugation at 500xg for 1 min at 4°C. An aliquot of the sample containing 10 mg of protein extract was then added tothe microcentrifuge tube containing the equilibrated GFP-Trap agarose beads and incubated on a rotating shaker at 4°C overnight. The beads with bound protein were collected by centrifugation at 500xg for 1 min at 4°C and then resuspended in 500 μl of ice-cold extraction buffer, followed by centrifugation at 500xg for 1 min at 4°C. This step was repeated once more. The bound protein was liberated by adding 30 μl of 5x Laemmli SDS-PAGE sample buffer [54] and heating at 95°C for 5 minutes, followed by centrifugation at 500xg for 5 min at 4°C. The supernatants were removed, separated using 10% SDS-PAGE and analyzed by immunoblot using anti-GFP or anti-S tag antibodies as described above.

## Results

### Expression of *cna-1* and *cnb-1* under Control of the *tcu-1* Promoter

Previous work in *N*. *crassa* has suggested that the catalytic subunit of calcineurin, *cna-1*, is an essential gene [28], while the regulatory subunit, *cnb-1*, is not [30]. In order to address the putative essential nature of *cna-1*, we produced strains that express *cna-1* under the control of a regulatable promoter. We included *cnb-1* in these studies as a nonessential gene that would serve as a control for the method. We took advantage of a recently developed system using the copper-regulated *tcu-1* promoter [33] in *N*. *crassa*, inserting the constructs at the *pan-2* locus [34]. The *tcu-1* promoter is expressed in limiting copper II, which can be imposed through addition of a copper chelator such as BCS to the growth medium [33].

**Fig 1:**
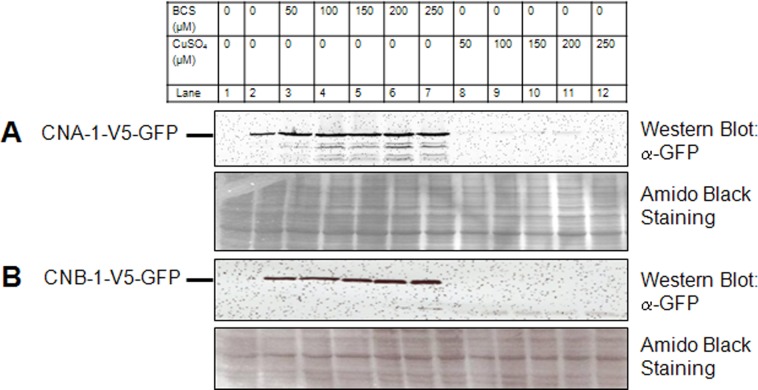
Copper-regulated expression of CNA-1 and CNB-1 under control of the *tcu-1* promoter. The effect of BCS or copper sulfate on P_*tcu-1*_ driven expression of CNA-1 from the *∆cna-1*::*hph; ∆pan-2*::P_*tcu-1*_::*cna-1*::*v5*::*gfp; mat a* (540) strain **(A)**, and CNB-1 from the *∆cnb-1*::*hph; ∆pan-2*::P_*tcu-1*_::*cnb-1*::*v5*::*gfp; mat a* (597) strain **(B)** was determined (lanes 2–12) after growth of strains in liquid medium under the indicated conditions for 22 h. Protein isolated from the wild type strain (FGSC 2489) was used as control (lane 1). Protein extracts were prepared and samples containing 50 μg of total protein were analyzed by Western blot using rabbit anti-GFP antibody as indicated in the Materials and Methods. The solid lines indicate positions of CNA-1::V5::GFP (~93 kDa) and CNB-1::V5::GFP (~48 kDa) proteins. The amido black staining of the membrane, shown in the lower panel, was done to demonstrate equal protein loading.

To verify that expression of the CNA-1 and CNB-1 proteins was regulated by copper availability, the strains were grown in minimal medium (VM) supplemented with various amounts of BCS and/or copper sulfate and protein expression was examined using Western analysis. We included a wild type strain lacking the GFP-tagged constructs for CNA-1 and CNB-1 as a negative control for the presence of the two proteins (Fig 1). The CNA-1 and CNB-1 proteins were abundantly expressed in the medium supplemented with BCS, but not detectable in the presence of copper sulfate (Fig 1). These observations suggest that expression of the CNA-1 and CNB-1 proteins is controlled by copper availability in the medium, similar to results demonstrated for other heterologous proteins in the previous study [33].

### Repression of CNA-1 and CNB-1 Results in Severe Defects in Hyphal Growth and Aerial Hyphae Development

**Fig 2:**
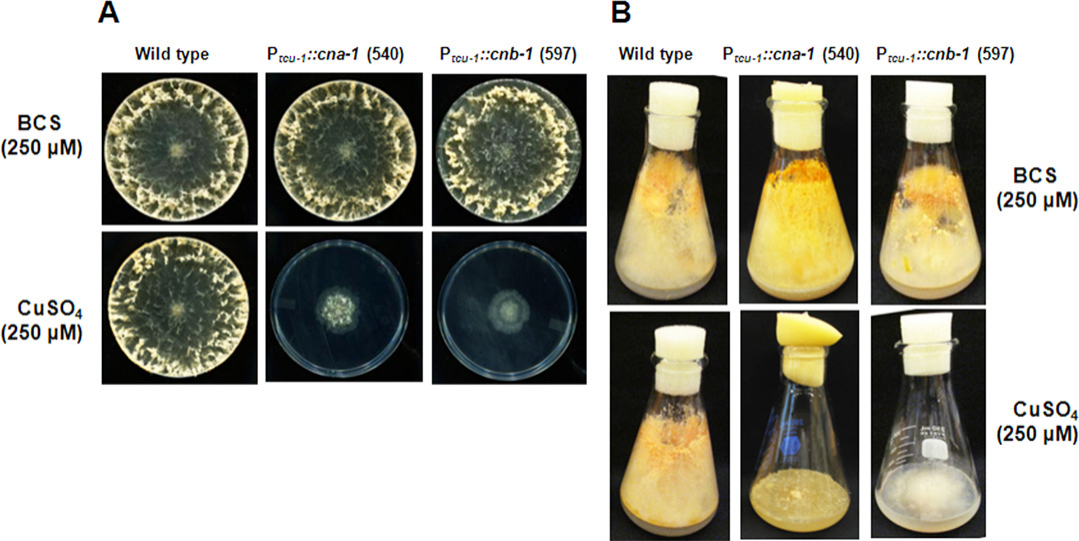
Effect of reduced expression of calcineurin on colony morphology. **(A) Colony morphology.** Wild type, *∆cna-1*::*hph; ∆pan-2*::P_*tcu-1*_::*cna-1*::*v5*::*gfp; mat a* (540), and *∆cnb-1*::*hph; ∆pan-2*::P_*tcu-1*_::*cnb-1*::*v5*::*gfp; mat a* (597) strains were cultured on VM plates supplemented with 250 μM of BCS (upper panel) or CuSO_4_ (lower panel). Strains were grown for one day at 30°C in the dark, followed by two days under constant light at room temperature and photographed using a Canon G10 camera. **(B) Morphology of flask cultures.** Cultures of wild type, *∆cna-1*::*hph; ∆pan-2*::P_*tcu-1*_::*cna-1*::*v5*::*gfp; mat a* (540), and *∆cnb-1*::*hph; ∆pan-2*::P_*tcu-1*_::*cnb-1*::*v5*::*gfp; mat a* (597) strains were grown in flasks containing VM agar medium supplemented with 250 μM of BCS (upper panel) or CuSO_4_ (lower panel). Strains were grown for three days at 30°C in dark and four days under light at room temperature and photographed using a Canon G10 camera.

**Fig 3:**
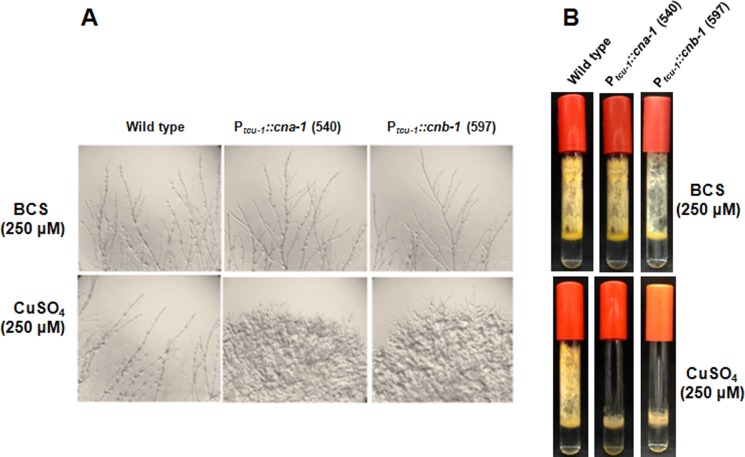
Effect of reduced expression of calcineurin on hyphal growth. **(A) Hyphal morphology**. The wild type, *∆cna-1*::*hph; ∆pan-2*::P_*tcu-1*_::*cna-1*::*v5*::*gfp; mat a* (540), and *∆cnb-1*::*hph; ∆pan-2*:: P_*tcu-1*_::*cnb-1*::*v5*::*gfp; mat a* (597) strains were cultured on VM medium supplemented with 250 μM of BCS (upper panel) or CuSO_4_ (lower panel) for 24 h at 30°C and then photographed using a Canon G10 camera with an Olympus SZX9 stereo microscope. **(B) Aerial hyphae growth.** Wild type, *∆cna-1*::*hph; ∆pan-2*:: P_*tcu-1*_::*cna-1*::*v5*::*gfp; mat a* (540), and *∆cnb-1*::*hph; ∆pan-2*::P_*tcu-1*_::*cnb-1*::*v5*::*gfp; mat a* (597) strains in VM liquid medium supplemented with 250 μM of BCS (upper panel) or CuSO_4_ (lower panel). Strains were grown for three days at 30°C in dark and four days under light at room temperature and photographed using a Canon G10 camera.

To test the requirement for CNA-1 and CNB-1 during growth and development of *N*. *crassa*, we determined the effect of copper availability on morphology of the strains with *cna-1* or *cnb-1* under the control of the *tcu-1* promoter (Figs 2 and 3). The 540 and 597 strains exhibited normal growth on agar medium supplemented with BCS; however, severe impairment was observed on the medium supplemented with excess copper sulfate (Fig 2). The average colony diameter of the two strains was reduced ~74% in the medium supplemented with excess BCS vs. copper sulfate (Table 3). Furthermore, consecutive transfers of the strains on the repressing medium continued to show severe growth impairment, but growth was reverted back to normal after inoculation of the strains on medium containing BCS (data not shown). Therefore, the growth impairment of strains 540 and 597 by excess copper sulfate was reversible. In addition, the hyphal growth of 540 and 597 was abnormal, with bulged hyphal tips produced in the presence of excess copper sulfate (Fig 3A). Moreover, aerial hyphae production in the 540 and 597 strains was greatly reduced in the repressing high copper medium (Fig 3B). These results indicate thatCNA-1 and CNB-1 play an important role in regulating vegetative growth and hyphal development in *N*. *crassa*.

**Table 3:** Average growth rate.

Strain	Average growth rate (cm h^-1^)	
	VM+pantothenate+BCS	VM+pantothenate+CuSO_4_
Wild type: 74-OR23-IVA	0.377 ±0.031	0.380 ±0.025
Strain 540: *∆cna-1*::*hph; ∆pan-2*::P_*tcu-1*_::*cna-1*::*V5*::*gfp; mat a*	0.391 ±0.006	0.103 ±0.005
Strain 597: *∆cnb-1*::*hph; ∆pan-2*::P_*tcu-1*_::*cnb-1*::*V5*::*gfp; mat a*	0.303 ±0.011	0.079 ±0.004

### Reduced Expression of CNA-1 and CNB-1 Affects Female Sexual Fertility

To determine possible roles for CNA-1 and CNB-1 in sexual fertility, we analyzed crosses involving strains 540 and 597 on sexual crossing medium (SCM) supplemented with either BCS or excess copper. In the inducing BCS medium, the crosses involving strains 540 and 597 as female parents with a wild type male parent were fertile (produced thousands of ascospores; Table 4), with strain 540 slightly less fertile than the wild type control (Table 4). Under repressing conditions, crosses involving strain 540 as the female parent with a wild type male produced peithecia-like structures; however, when these were dissected, they were devoid of asci and ascospores. Thus strain 540 is female-sterile in excess copper sulfate, indicating a strict requirement for CNA-1 in female structures during the sexual cycle (Table 4). The need for CNB-1 appeared to be not as critical, as crosses with strain 597 as the female parent produced an intermediate number of ascospores relative to wild type (Table 4). Crosses involving wild type as the female parent and strain 540 or 597 as the male parent in the SCM supplemented with only pantothenate (Table 4) or minimal SCM were intermediate and fertile, respectively. Therefore, of the two subunits of calcineurin, CNA-1 seems to be essential for female fertility and may also influences male fertility.

**Table 4:** Sexual cycle phenotypes.

Female Parent	Male Parent	Supplement	Phenotype
Wild type *mat A*	Wild type *mat a*	Pantothenate+BCS	Fertile, tens of thousands of ascospores
Wild type *mat a*	Wild type *mat A*	Pantothenate+BCS	Fertile, tens of thousands of ascospores
Strain 540 *mat a*	Wild type *mat A*	Pantothenate+BCS	Intermediate, few thousands of ascospores
Strain 597 *mat a*	Wild type *mat A*	Pantothenate+BCS	Fertile, tens of thousands of ascospores
Wild type *mat A*	Wild type *mat a*	Pantothenate+CuSO_4_	Fertile, tens of thousands of ascospores
Wild type *mat a*	Wild type *mat A*	Pantothenate+CuSO_4_	Fertile, tens of thousands of ascospores
Strain 540 *mat a*	Wild type *mat A*	Pantothenate+CuSO_4_	Sterile, no ascospores
Strain 597 *mat a*	Wild type *mat A*	Pantothenate+CuSO_4_	Intermediate, few hundreds of ascospores
Wild type *mat A*	Wild type *mat a*	Pantothenate	Fertile, tens of thousands of ascospores
Wild type *mat a*	Wild type *mat A*	Pantothenate	Fertile, tens of thousands of ascospores
Wild type *mat A*	Strain 540 *mat a*	Pantothenate	Intermediate, few thousands of ascospores
Wild type *mat A*	Strain 540 *mat a*	Pantothenate+BCS	Intermediate, few thousands of ascospores
Wild type *mat A*	Strain 597 *mat a*	Pantothenate	Fertile, tens of thousands of ascospores

### *cnb-1* ^RIP^-Mutants Possess Defects in Growth and Fertility

Due to the lack of a *∆cnb-1*::*hph mat A* strain, it was necessary to perform two crosses using the *mat a* transformants with the *tcu-1* promoted version of *cnb-1* in order to create the final *∆cnb-1*::*hph; ∆pan-2*:: P_*tcu-1*_::*cnb-1*::*v5*::*gfp* strains (see Materials and Methods). This meant that the *cnb-1* ORF in these strains might be subjected to RIP during the second cross. For this reason, it was necessary to sequence the *cnb-1* ORF in the final cross progeny. We determined that strains 599, 600 and 602 had accrued RIP-induced mutations spread throughout the *cnb-1* gene, including the Ca^2+^-binding domains (Materials and Methods; S1 Fig). Several nonsynonymous substitutions were also identified in the *cnb-1*^RIP^ allele of these strains (S1 Fig). Strain 599 contains a M31I (ATG →ATA codon change) mutation in the EF1 domain. Strain 600 contains M31I (ATG →ATA codon change) mutation in the EF1 domain, D103N (GAC→AAC codon change), D107N (GAC→AAC codon change) and M121I (ATG→ATA codon change) mutations in the EF hand 3. Strain 602 contains M31I (ATG →ATA codon change) mutation in the EF1 domain, and D105N (GAC→AAC codon change) mutation in the EF hand loop 3, respectively.

Using Western analysis, we determined that the CNB-1 protein was expressed in the three RIP mutant strains under inducing conditions (S2 Fig). This result indicated that the RIP mutant proteins were translated and stable. However, phenotypic analysis demonstrated that these strains exhibited defects in colony morphology, growth rate, and aerial hyphae development in medium supplemented with excess copper (S3 Fig; S1 Table). The colony morphology of these strains was defective even in the inducing medium, and the *cnb-1*^RIP^ (602) mutant showed a severe growth defect (S3 Fig; S1 Table). Furthermore, aerial hyphae development in the *cnb-1*^RIP^ (600) and *cnb-1*^RIP^ (602) mutants was defective even in inducing medium (S3 Fig). In addition, in the inducing medium, when used as a female parent with the wild type as male parent, strain *cnb-1*^RIP^ (599) exhibited an intermediate phenotype, strain *cnb-1*^RIP^ (600) was fertile, and strain *cnb-1*^RIP^ (602) was sterile (S2 Table). In the repressing medium, when used as a female parent with the wild type as male parent, the *cnb-1*^RIP^ (599) and *cnb-1*^RIP^ (600) strains were fertile; while strain *cnb-1*^RIP^ (602) was sterile (S2 Table). All three *cnb-1*^RIP^-mutants were fertile when used as males with a wild type female parent in SCM supplemented with pantothenate or minimal SCM (S2 Table). Therefore, *cnb-1*^RIP^ (599) and *cnb-1*^RIP^ (600) mutants were fully fertile either as a male or female parent; however, *cnb-1*^RIP^ (602) mutant was sterile as a female parent in both inducing and repressing medium. Thus, the different RIP-induced alterations in the *cnb-1* gene of *cnb-1*^RIP^ mutants (599, 600, and 602) result in phenotypic differences.

### CNA-1 and CNB-1 Form a Complex

To test whether CNA-1 and CNB-1 form a complex *in vivo*, we produced *N*. *crassa* strains that expressed RFP and GFP-tagged versions of these proteins from the *inl* and *pan-2* loci, respectively (see Materials and Methods). We isolated homokaryotic stains expressing CNA-1::S-tag::RFP (strain 767; requires inositol) and CNB-1::V5::GFP (strain 727; requires pantothenate) tagged proteins.The CNA-1 and CNB-1 proteins contain 562 and 174 amino acid residues having predicted molecular weights of 64.5 and 19.8 kDa, respectively. The combined predicted molecular weights of the tagged proteins, CNA-1::S-tag::RFP and CNB-1::V5::GFP, are, respectively, 92.6 and 48.4 kDa. Expression of CNA-1 and CNB-1 tagged proteins of the correct size was verified using Western analysis (data not shown).

**Fig 4:**
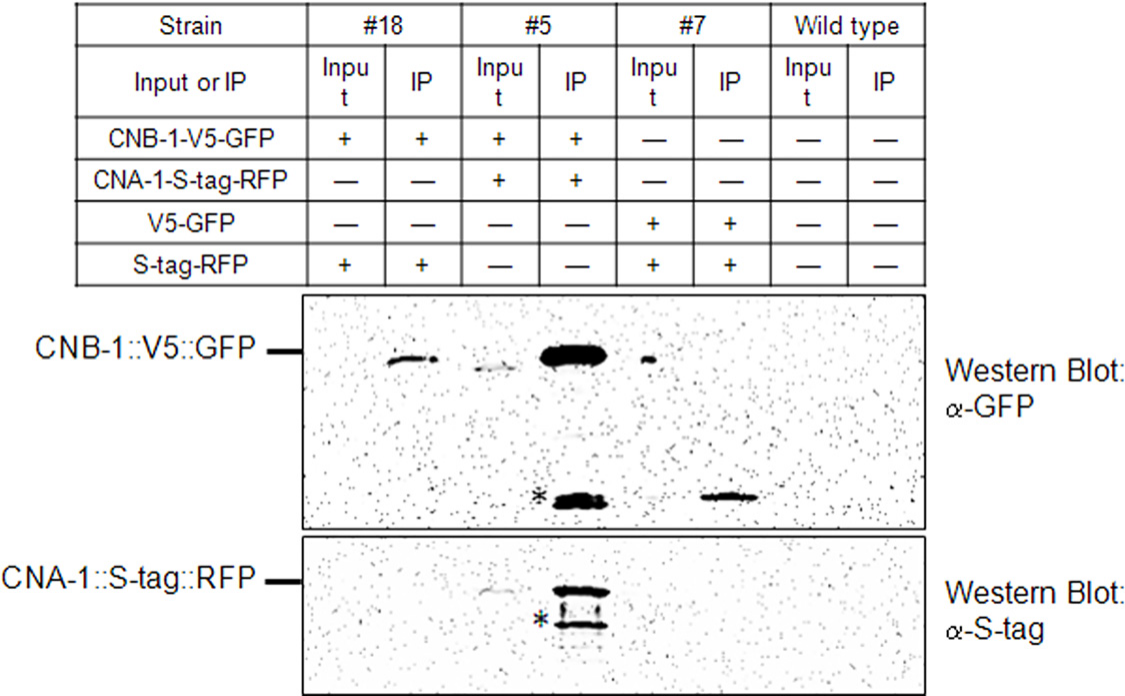
Co-immunoprecipitation of CNA-1 and CNB-1 proteins. The indicated heterokaryons (#5, 7, and 18), and the wild type strain were grown for 16 h in submerged culture as described in the Materials and Methods. The soluble fraction was isolated and immunoprecipitated using anti-GFP antibody coupled to agarose beads. Samples of whole cell lysates (Input) and immunoprecipitated proteins (IP) were subjected to Western blot analysis using GFP (top), and S-tag (bottom) antibodies as described in Materials and Methods. The line indicates position for bands of the CNB-1::V5::GFP (~48 kDa; Top Blot) and CNA-1::S-tag::RFP (~ 93 kDa; Bottom blot) proteins. The asterisk is pointing to a smaller molecular weight molecule possibly resulted from proteolytic cleavage of CNB-1::V5::GFP and CNA-1::S-tag::RFP.

The tagged CNA-1 and CNB-1 proteins were brought together in the same cytoplasm through formation of forced heterokaryons between strains 767 and 727 (Table 1). The two strains were co-inoculated on Vogel’s minimal agar medium lacking inositol and pantothenate, conditions under which only the heterokaryon will grow. Control heterokaryons contained the CNB-1-GFP strain and a strain only expressing RFP (Fig 4; Table 1). Heterokaryons were cultivated in liquid medium and protein extracts were prepared. Extracts were treated with anti-GFP antibody coupled to agarose beads, and the immunoprecipitated proteins were analyzed by immunoblot using anti-GFP and anti-S-tag (detects S-tag-RFP-tagged protein) antibodies (Fig 4). The results demonstrated that CNB-1-V5-GFP was immunoprecipitated in all strains where it was present using the anti-GFP beads (Fig 4). Importantly, the ~93 kDa CNA-1-S-tag-RFP protein was visible in the input from the CNB-1-GFP+CNA-1-RFP strain and also co-immunoprecipitated with CNB-1-GFP (Fig 4). In control reactions, CNA-1-S-tag-RFP was not co-immunoprecipitated in strains expressing only V5-GFP, and S-tag-RFP was not co-immunoprecipitated using CNB-1-V5-GFP (Fig 4), showing that the fusion proteins do not interact with the fluorescent protein tags. In addition, microscopy analysis revealed co-localization of the tagged CNA-1 and CNB-1 proteins, which supports *in vivo* interaction between two calcineurin subunits (S4 Fig). These results indicate that CNA-1 and CNB-1 form a complex in *N*. *crassa*.

## Discussion

Calcineurin is the only protein phosphatase that depends on both Ca^2+^ and CaM for its activity [1, 2]. Calcineurin consists of two subunits, a catalytic A and a regulatory B that are, respectively, encoded by the *cna-1* and the *cnb-1* genes in *N*. *crassa*. We studied here the cellular roles of calcineurin via its heterologous expression under the conditional *tcu-1* promoter. In this system, expression of the heterologous gene cloned under the P_*tcu-1*_ is controlled by copper availability in a rapid and stable manner [32, 33]. Using this system, we found that both CNA-1 and CNB-1 were required for normal vegetative and hyphal growth, and sexual development of *N*. *crassa*. Previous work using *cna-1* antisense RNA also showed its essential role in normal hyphal growth, morphology, and maintenance of the apical tip-high Ca^2+^ gradient [28]. Moreover, immunosuppressive fungicidal drugs cyclosporin A (CsA) and FK506 were shown to inhibit hyphal growth of *N*. *crassa* by inactivating calcineurin. In addition, studies using the insertional and RIP mutants of the *cnb-1* suggested its requirement for normal vegetative growth and a possible role in repressing the asexual developmental program in *N*. *crassa* [30]. Calcineurin is also required for normal hyphal growth in fungal pathogens in *C*. *neoformans*, *A*. *fumigatus*, *M*. *oryzae*, and *U*. *maydis*, suggesting a general role of calcineurin in governing hyphal growth [21, 22, 24, 25, 27, 55, 56].

Calcineurin also plays a role in fertility and stress response pathways in various organisms. In the model nematode *C*. *elegans*, calcineurin regulates fertility [57]. In addition, calcineurin is also reported to play a role in stress and pheromone response pathways in in other fungi, such as *S*. *cerevisiae*, *Schizosaccharomyces pombe*, *C*. *albicans*, and *C*. *neoformans* [15–18, 23, 58]. We have demonstrated that calcineurin subunit A plays an essential role during sexual development in *N*. *crassa*. In *N*. *crassa*, the sexual phase starts with fertilization between two opposite mating type nuclei in limited nitrogen and nutrient starvation, a stress condition. In addition, sexual development in *N*. *crassa* normally proceeds with development of protoperithecia and then perithecia, but the perithecia were empty under repressing conditions for the CNA-1 strain (540) as the female parent, suggesting that this protein must be important for some aspect of meiosis or ascospore development. Moreover, CNA-1 strain as the male parent was less productive, which could indicate a possible involvement of this protein in initial steps of mating. In addition, the CNB-1 appeared to be not as critical, and thus, that the phenotypes of the strains CNA-1 (540) and CNB-1 (597) in sexual development differ, suggesting that CNA-1 may have novel functions independent of CNB-1. In this study, we also isolated RIP mutated alleles of *cnb-1*. Among the non-synonymous substitutions, the *cnb-1*^RIP^ (599) allele contains M31I alteration in the EF1. Similarly, the *cnb-1*^RIP^ (600) allele contains M31I in the EF1, and D103N, D107N, and M121I alterations in the EF3. In addition, the *cnb-1*^RIP^ (602) allele contains M31I and D105N alterations in the EF1 and EF3, respectively. The EF hand contains a helix-loop-helix structural unit known as EF hand or Ca^2+^ binding loop that contains characteristic 12 amino acid residues involved in the Ca^2+^ binding [59]. Ca^2+^ is coordinated directly by oxygen atoms provided by the side chains of residues at positions 1(+*x*), 3(+*y*), 5(+*z*) and 12 (*-z*) [59]. The residues D103 and D107 constitute the 1(+*x*) and 5(+*z*) position of the EF hand loop 3, respectively [59]. The Ca^2+^ coordinating residues D103 and D107 are also conserved among the CNB1 from different fungi, Drosophila, and mammals (data not shown). Therefore, the D103N and D107N alterations in the *cnb-1*^*RIP*^ (600) allele are expected to cause a defect in Ca^2+^ co-ordination in this mutant, and this could explain the growth defect of the *∆cnb-1*::*hph; ∆pan-2*::P_*tcu-1*_::*cnb-1* ^RIP^::*v5*::*gfp; mat a* (600) strain even under inducing conditions (S3 Fig). EF3 of the *cnb-1*^RIP^ (600) allele contains RIP mutations both inside and outside of the EF hand loop. The major alteration found in the *cnb-1*^RIP^ (602) allele was D105N in the EF3. The D105 residue is the coordinate 3(+*y*) involved in Ca^2+^ binding through the oxygen atoms of aspartate [59]. Therefore, the *cnb-1*^RIP^ (602) allele contains an EF3 domain with defective Ca^2+^ binding ability and this could explain the growth and fertility defects of the *∆cnb-1*::*hph; ∆pan-2*::P_*tcu-1*_::*cnb-1* ^RIP^::*v5*::*gfp; mat a* (602) strain in both inducing and repressing medium (S3 Fig; S2 Table). The *cnb-1*^RIP^ (602) was sterile under both inducing and repressing medium, while the wild type allele was fertile. This suggests that very little wild type CNB-1 protein is necessary to support female fertility, while the *cnb-1*^RIP^ (602) mutation impairs function so that even high levels of protein do not support female fertility. Thus, this analysis has identified D103, D105, and D107 as critical amino acid residues in the EF3 domain of *cnb-1*.

Interaction between the recombinant forms of the bovine A and rat B subunits of calcineurin, expressed in *E*. *coli*, was demonstrated *in vitro* [60]. The interaction between the calcineurin A and B subunits were shown in *S*. *cerevisiae* using the yeast two-hybrid system [61]. We demonstrated *in vivo* interaction between tagged versions of CNA-1 and CNB-1 by co-immunoprecipitation and co-localization studies which indicates formation of a complex of these two proteins in *N*. *crassa* (Fig 4; S4 Fig).

In this study, we showed the cellular roles of calcineurin in growth, development, and fertility in *N*. *crassa*. Moreover, we demonstrated *in vivo* interaction between the calcineurin subunits, CNA-1 and CNB-1, and identified three critical amino acid residues in CNB-1. Future work will identify the target transcription factors of calcineurin in *N*. *crassa*.

## Supporting Information

S1 Fig RIP-introduced mutations of *cnb-1*. **A. Alignment of the wild type and *cnb-1***^**RIP**^ **alleles.** Mutations in the intronic regions, and synonymous and non-synonymous substitutionsare shown as tick, arrow head and solid arrow marks, respectively, above the alignment. **B. Alignment of the protein sequences of the CNB-1 and CNB-1**^**RIP**^ **proteins**. The positions of the EF-hand loops, EF1-EF4 (solid line) and the calcium binding regions (dotted line) are indicated above the sequence, as revealed by Uniprot analysis (http://www.uniprot.org/uniprot/P87072). The arrows indicate the altered amino acid residues. Conserved residues are indicated in black (100%), dark gray (>80%) and light gray (>60%).
(TIF)

S2 Fig Copper-regulated expression of CNB-1^RIP^proteins under the *tcu-1* promoter. Effects of BCS and copper sulfate on P_*tcu-1*_ driven expression of CNB-1 from the *cnb-1*^RIP^ strains. Samples containing 50 μg of total protein were analyzed by Western blot using rabbit anti-GFP antibody. An extract from untransformed wild type, grown in minimal VM, was used as a control. The expression level of CNB-1::V5::GFP in extracts from *∆cnb-1*::*hph;* P_*tcu-1*_::*cnb-1*::*v5*::*gfp*::*∆pan-2; mat a* strains (599, 600, and 602) treated with 250 μM of BCS (B) or copper (C) for 22 h, is indicated. The solid lines indicate positions of the CNB-1::V5::GFP protein of molecular weight (MW) ~ 48 in the strains 602, 600, and another band (possibly truncated) of ~25 kDa appeared in the lane for the strain 599 on the blot. The membrane was stained with amido black as a protein loading control (lower panel).
(TIF)

S3 Fig Growth phenotype of the *cnb-1*^RIP^ mutants. **A. Hyphal morphology.** Wild type and *cnb-1*^RIP^ mutants were grown for 24 h at 30°C on VM medium supplemented with 250 μM of BCS (upper panel) or CuSO_4_ (lower panel). **B. Colony morphology.** Wild type and the *cnb-1*^RIP^ mutants were cultured for 24 h at 30°C in dark and 48 h under light at room temperature on VM plates supplemented with 250 μM of BCS (upper panel) or CuSO_4_ (lower panel). **C. Growth of aerial hyphae.** Aerial hyphae of the wild type and the *cnb-1*^RIP^ mutants grown for 72 h at 30°C in dark and four days under light at room-temperature in VM liquid medium supplemented with 250 μM of BCS (upper panel) or CuSO_4_ (lower panel). All the strains were photographed using a Canon G10 camera.
(TIF)

S4 Fig Microscopy analysis for localization and *in vivo* interaction of the two caclineurin subunits. A heterokaryon (CNB-1GFP+CNA-1RFP#5, Table 1) expressing two fluorescent proteins, CNA-1::S-tag::RFP and CNB-1::V5::GFP, was analyzed using a confocal microscope to investigate the localization and *in vivo* interaction of the two caclineurin subunits. Forced heterokaryons were made same as described for co-immunoprecipitation analysis (see Materials and Methods), conidia were then isolated and inoculated in 5 ml liquid VM and incubated at 30°C for 6 h with shaking at 200 rpm. Germlings were analyzed using a Leica TCS SP8 (DMi8) confocal microscope with a 63x oil objective, 4x zoom, 1024x1024 pixels resolution, and scan speed of 400 Hz (Leica Microsystems CMS, GmbH, Germany). The CNA-1::S-tag::RFP and CNB-1::V5::GFP heterokaryons were visualized with the Hybrid Detection system (HyD) laser. Images were captured sequentially; RFP images were obtained with excitation at 543 nm and emission from 555–700 nm, and GFP images were obtained by excitation at 488 nm, with emission collected from 500–535 nm. DIC, RFP and GFP fluorescent, and merged images are shown in the columns from left to right, respectively. The arrowhead, asterisk, and solid arrow indicate CNA-1::S-tag::RFP, CNB-1::V5::GFP, and co-localization of the tagged calcineurin subunits, respectively. Scale bar = 5 μm.
(TIF)

S1 Table Average growth rate of colony diameter of the *cnb-1*^RIP^ mutants. (DOCX)

S2 Table Phenotype of crosses involving the *cnb-1*^RIP^ mutants. (DOCX)
