# Supplementary figures and images for: Calcineurin Subunits A and B Interact to Regulate Growth and Asexual and Sexual Development in Neurospora crassa
Source: PLoS One. 2016 Mar 28;11(3):e0151867. doi: 10.1371/journal.pone.0151867 (PMC4809485; doi:10.1371/journal.pone.0151867)

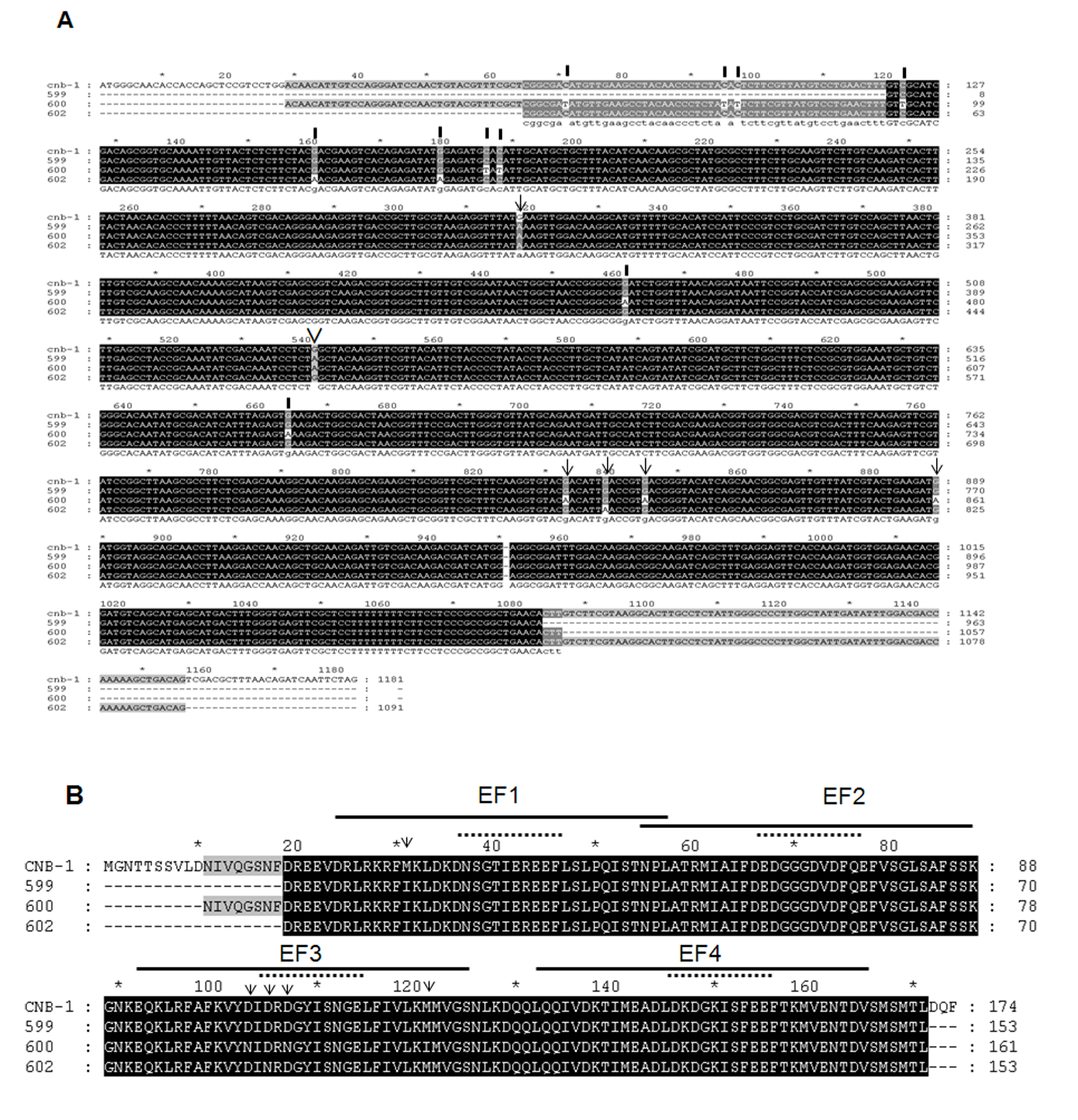

Supplement: S1 Fig — A. Alignment of the wild type and cnb-1RIP alleles. Mutations in the intronic regions, and synonymous and non-synonymous substitutionsare shown as tick, arrow head and solid arrow marks, respectively, above the alignment. B. Alignment of the protein sequences of the CNB-1 and CNB-1RIP proteins. The positions of the EF-hand loops, EF1-EF4 (solid line) and the calcium binding regions (dotted line) are indicated above the sequence, as revealed by Uniprot analysis (http://www.uniprot.org/uniprot/P87072). The arrows indicate the altered amino acid residues. Conserved residues are indicated in black (100%), dark gray (>80%) and light gray (>60%). (TIF) [file pone.0151867.s001.tif]

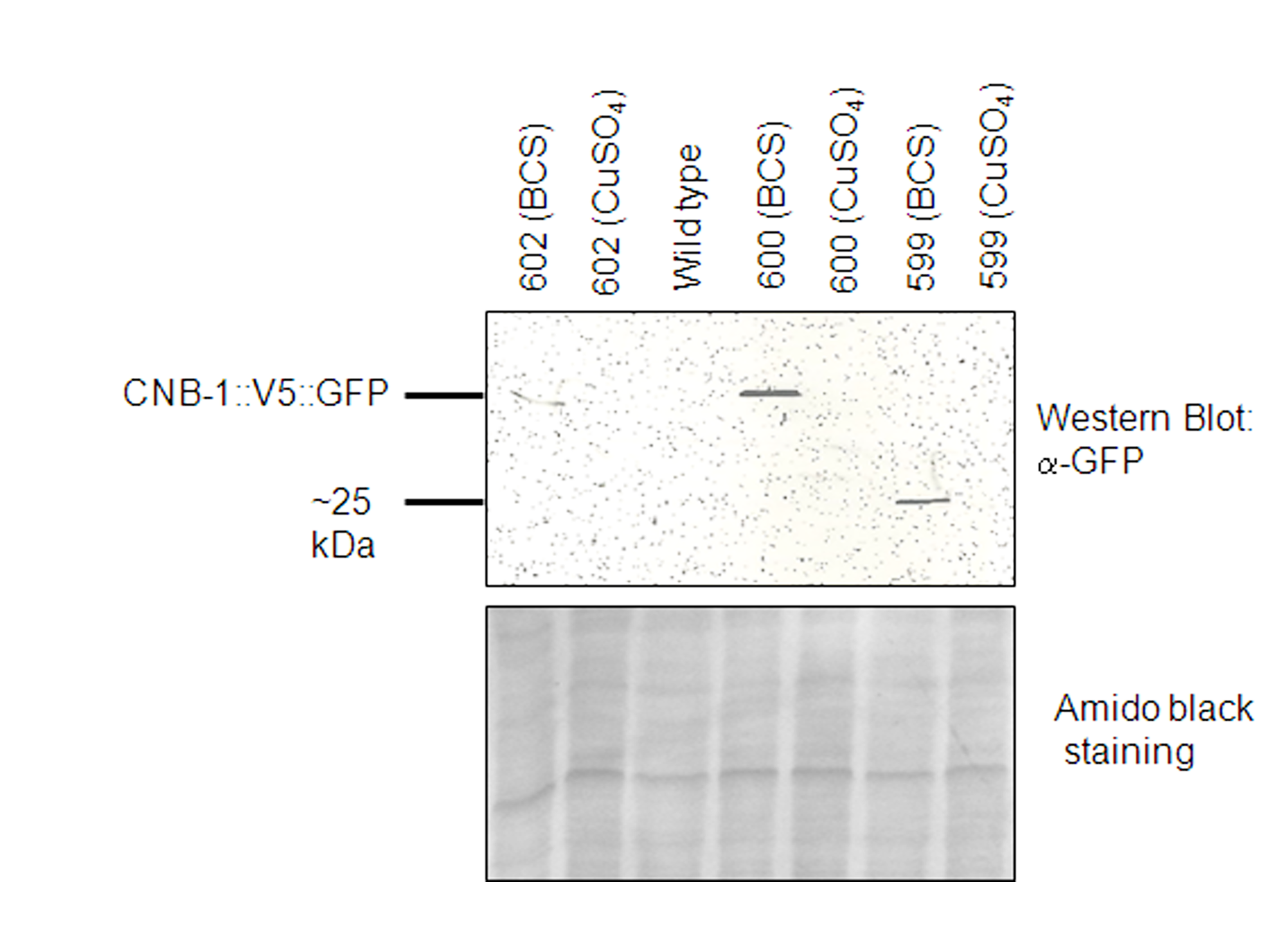

Supplement: S2 Fig — Effects of BCS and copper sulfate on Ptcu-1 driven expression of CNB-1 from the cnb-1RIP strains. Samples containing 50 μg of total protein were analyzed by Western blot using rabbit anti-GFP antibody. An extract from untransformed wild type, grown in minimal VM, was used as a control. The expression level of CNB-1::V5::GFP in extracts from ∆cnb-1::hph; Ptcu-1::cnb-1::v5::gfp::∆pan-2; mat a strains (599, 600, and 602) treated with 250 μM of BCS (B) or copper (C) for 22 h, is indicated. The solid lines indicate positions of the CNB-1::V5::GFP protein of molecular weight (MW) ~ 48 in the strains 602, 600, and another band (possibly truncated) of ~25 kDa appeared in the lane for the strain 599 on the blot. The membrane was stained with amido black as a protein loading control (lower panel). (TIF) [file pone.0151867.s002.tif]

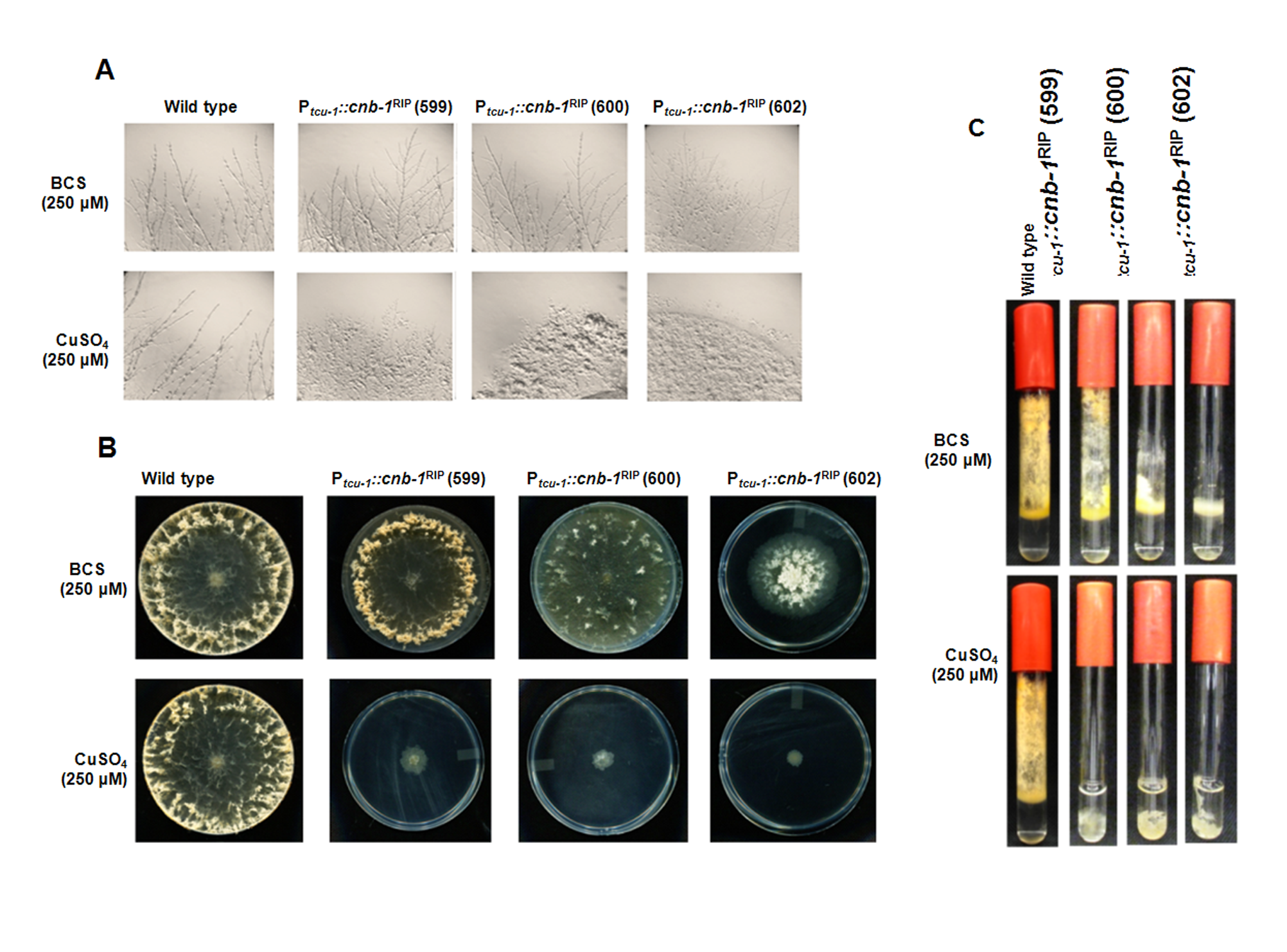

Supplement: S3 Fig — A. Hyphal morphology. Wild type and cnb-1RIP mutants were grown for 24 h at 30°C on VM medium supplemented with 250 μM of BCS (upper panel) or CuSO4 (lower panel). B. Colony morphology. Wild type and the cnb-1RIP mutants were cultured for 24 h at 30°C in dark and 48 h under light at room temperature on VM plates supplemented with 250 μM of BCS (upper panel) or CuSO4 (lower panel). C. Growth of aerial hyphae. Aerial hyphae of the wild type and the cnb-1RIP mutants grown for 72 h at 30°C in dark and four days under light at room-temperature in VM liquid medium supplemented with 250 μM of BCS (upper panel) or CuSO4 (lower panel). All the strains were photographed using a Canon G10 camera. (TIF) [file pone.0151867.s003.tif]

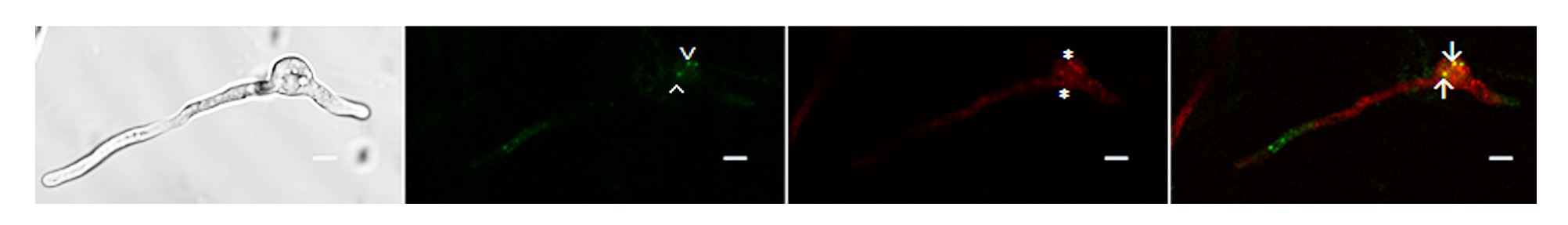

Supplement: S4 Fig — A heterokaryon (CNB-1GFP+CNA-1RFP#5, Table 1) expressing two fluorescent proteins, CNA-1::S-tag::RFP and CNB-1::V5::GFP, was analyzed using a confocal microscope to investigate the localization and in vivo interaction of the two caclineurin subunits. Forced heterokaryons were made same as described for co-immunoprecipitation analysis (see Materials and Methods), conidia were then isolated and inoculated in 5 ml liquid VM and incubated at 30°C for 6 h with shaking at 200 rpm. Germlings were analyzed using a Leica TCS SP8 (DMi8) confocal microscope with a 63x oil objective, 4x zoom, 1024x1024 pixels resolution, and scan speed of 400 Hz (Leica Microsystems CMS, GmbH, Germany). The CNA-1::S-tag::RFP and CNB-1::V5::GFP heterokaryons were visualized with the Hybrid Detection system (HyD) laser. Images were captured sequentially; RFP images were obtained with excitation at 543 nm and emission from 555–700 nm, and GFP images were obtained by excitation at 488 nm, with emission collected from 500–535 nm. DIC, RFP and GFP fluorescent, and merged images are shown in the columns from left to right, respectively. The arrowhead, asterisk, and solid arrow indicate CNA-1::S-tag::RFP, CNB-1::V5::GFP, and co-localization of the tagged calcineurin subunits, respectively. Scale bar = 5 μm. (TIF) [file pone.0151867.s004.tif]
