# Supplementary material for: Calcineurin Subunits A and B Interact to Regulate Growth and Asexual and Sexual Development in Neurospora crassa
Source: PLoS One. 2016 Mar 28;11(3):e0151867. doi: 10.1371/journal.pone.0151867 (PMC4809485; doi:10.1371/journal.pone.0151867)
Supplement: S1 Table — (DOCX) [file pone.0151867.s005.docx]

**S1 Table. Average growth rate of colony diameter of the *cnb-1*^RIP^ mutants.**

| Strain | Average growth rate (cm h^-1^) | |
| --- | --- | --- |
|  | **Medium** | |
|  | VM+Pan+BCS | VM+Pan+CuSO_4_ |
| Wild type: 74-OR23-IVA | 0.377 ±0.031 | 0.380 ±0.025 |
| *cnb-1*^RIP^ (strain 599) | 0.278 ±0.008 | 0.065 ±0.075 |
| *cnb-1*^RIP^ (strain 600) | 0.319 ±0.009 | 0.051 ±0.002 |
| *cnb-1*^RIP^ (strain 602) | 0.121 ±0.002 | 0.037 ±0.001 |
