# Supplementary material for: Calcineurin Subunits A and B Interact to Regulate Growth and Asexual and Sexual Development in Neurospora crassa
Source: PLoS One. 2016 Mar 28;11(3):e0151867. doi: 10.1371/journal.pone.0151867 (PMC4809485; doi:10.1371/journal.pone.0151867)
Supplement: S2 Table — (DOCX) [file pone.0151867.s006.docx]

**S2 Table. Phenotype of crosses involving the *cnb-1*^RIP^ mutants.**

| Female Parent | Male Parent | Supplement in SCM | Phenotype |
| --- | --- | --- | --- |
| Wild type *mat* *A* | Wild type *mat* *a* | Pantothenate +BCS | Fertile, tens of thousands of ascospores |
| Wild type *mat* *a* | Wild type *mat* *A* | Pantothenate +BCS | Fertile, tens of thousands of ascospores |
| Strain 599 *mat* *A* | Wild type *mat* *a* | Pantothenate +BCS | Intermediate, few hundreds of ascospores |
| Strain 600 *mat* *A* | Wild type *mat* *a* | Pantothenate +BCS | Fertile, tens of thousands of ascospores |
| Strain 602 *mat* *A* | Wild type *mat* *a* | Pantothenate +BCS | Sterile, no ascospores |
| Wild type *mat* *A* | Wild type *mat* *a* | Pantothenate +CuSO_4_ | Fertile, tens of thousands of ascospores |
| Wild type *mat a* | Wild type *mat* *A* | Pantothenate +CuSO_4_ | Fertile, tens of thousands of ascospores |
| Strain 599 *mat* *A* | Wild type *mat* *a* | Pantothenate +CuSO_4_ | Fertile, few of thousands (~5000) of ascospores |
| Strain 600 *mat* *A* | Wild type *mat a* | Pantothenate +CuSO_4_ | Fertile, tens of thousands of ascospores |
| Strain 602 *mat* *A* | Wild type *mat* *a* | Pantothenate +CuSO_4_ | Sterile, no ascospores |
| Wild type *mat A* | Wild type *mat* *a* | Pantothenate | Fertile, tens of thousands of ascospores |
| Wild type *mat* *a* | Wild type *mat* *A* | Pantothenate | Fertile, tens of thousands of ascospores |
| Wild type *mat* *a* | Strain 599 *mat* *A* | Pantothenate | Fertile, tens of thousands of ascospores |
| Wild type *mat* *a* | Strain 600 *mat* *A* | Pantothenate | Fertile, tens of thousands of ascospores |
| Wild type *mat* *a* | Strain 602 *mat* *A* | Pantothenate | Fertile, tens of thousands of ascospores |
| Wild type *mat* *A* | Wild type *mat* *a* | None | Fertile, tens of thousands of ascospores |
| Wild type *mat* *a* | Wild type *mat* *A* | None | Fertile, tens of thousands of ascospores |
| Wild type *mat* *a* | Strain 599 *mat* *A* | None | Fertile, tens of thousands of ascospores |
| Wild type *mat* *a* | Strain 600 *mat* *A* | None | Fertile, tens of thousands of ascospores |
| Wild type *mat* *a* | Strain 602 *mat* *A* | None | Fertile, tens of thousands of ascospores |
